# Multidrug-Resistant Serratia marcescens Cavitary Pneumonia in an HIV Patient: A Case Report

**DOI:** 10.7759/cureus.99765

**Published:** 2025-12-21

**Authors:** Laxman Wagle, Prachi Bhanvadia, Sishir Poudel, Anuj Timshina, Sandeep Regmi

**Affiliations:** 1 Internal Medicine, Ascension Saint Agnes Hospital, Baltimore, USA; 2 Internal Medicine, B.P. Koirala Institute of Health Sciences, Dharan, NPL

**Keywords:** cavitary pneumonia, hiv, immunocompromised host, multidrug-resistant bacteria, opportunistic infection, septic shock, serratia marcescens

## Abstract

*Serratia marcescens* is an opportunistic gram-negative bacillus with both intrinsic and acquired resistance mechanisms, making it a challenging pathogen in immunocompromised individuals. We report a fatal case of cavitary pneumonia caused by multidrug-resistant *S. marcescens* in a 42-year-old HIV-positive man with chronic comorbidities, who presented with acute hypoxic respiratory failure and septic shock. Imaging revealed a large cavitary lesion in the right upper lobe, and cultures from blood, urine, and respiratory secretions confirmed *S. marcescens* with broad-spectrum antibiotic resistance. Despite aggressive medical management, including broad-spectrum antimicrobials, vasopressor support, bronchoscopy, and renal replacement therapy, the patient’s condition deteriorated, resulting in death. This case underscores the rare but severe pulmonary manifestation of *S. marcescens* in immunocompromised hosts, emphasizing the importance of early recognition, infection control, and targeted antimicrobial therapy in managing such high-risk infections.

## Introduction

*Serratia marcescens*, a gram-negative facultative anaerobic bacterium, is known for its intrinsic resistance to multiple antibiotics and its ability to acquire additional resistance, making infections caused by it particularly difficult to treat. It can cause a wide range of clinical diseases, including central nervous system infections such as meningitis, urinary tract infections, pneumonia, and other respiratory tract diseases, bloodstream infections such as endocarditis, and various wound infections [1]. In a retrospective eight-year study of 2,398 HIV-infected patients, *S. marcescens* infections were identified in 17 cases, including pneumonia in six patients [2]. We present a fatal case of *S. marcescens* cavitary pneumonia in an HIV patient.

## Case presentation

A 42-year-old man with a medical history of HIV (on dolutegravir/emtricitabine/tenofovir, last CD4 count 181 nine months prior), chronic sacral osteomyelitis, multidrug-resistant bacteremia (methicillin-resistant Staphylococcus epidermidis three months prior, Pseudomonas aeruginosa nine months prior), pulmonary fibrosis, and overactive bladder with a chronic indwelling Foley catheter presented with progressively worsening shortness of breath. On arrival at the emergency department, he was acutely hypoxic and rapidly deteriorated, requiring urgent intubation. He was found to be in septic shock, with a blood pressure of 72/42 mmHg and a mean arterial pressure of 52 mmHg, necessitating vasopressor support and admission to the intensive care unit (ICU) for close monitoring and management.

Initial laboratory investigations were notable for leukocytosis with a white blood cell count of 30.6 × 10⁹/L (reference range: 4.5-11.0× 10⁹/L), predominantly neutrophilic with bandemia. He also had acute kidney injury (creatinine 5.2 mg/dL (reference range: 0.7-1.3 mg/dL), BUN 57 mg/dL (reference range: 8-24 mg/dL) and severe metabolic acidosis on arterial blood gas (pH 7.05 (reference range: 7.35-7.45), bicarbonate 7 mmol/L (reference range: 22-26 mmol/L), and lactate 1.7 mmol/L (reference range: <2 mmol/L)).

**Figure 1:**
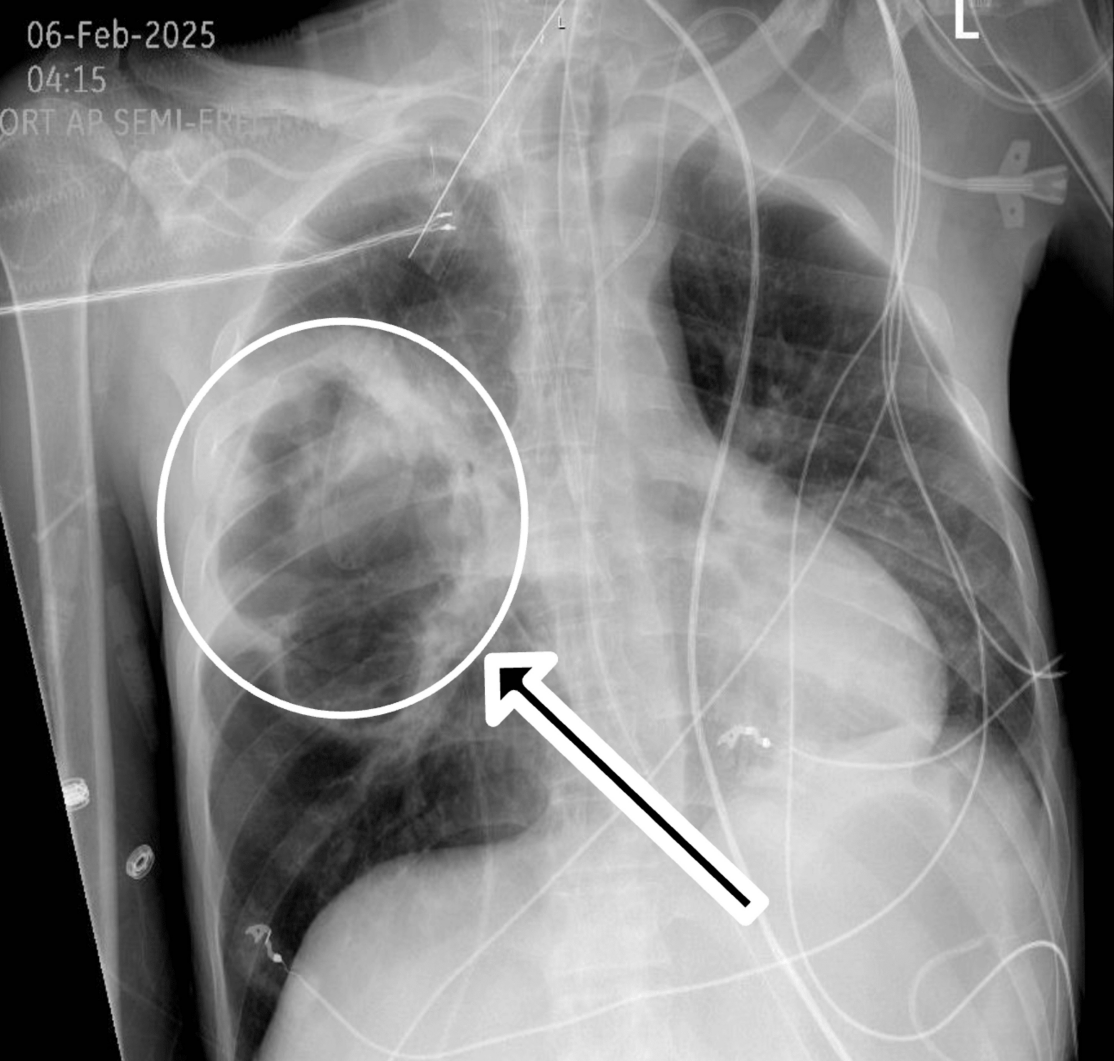
Chest X-ray showing large approximately 12 cm cavitation in the right lung (white circle and black arrow)

**Figure 2:**
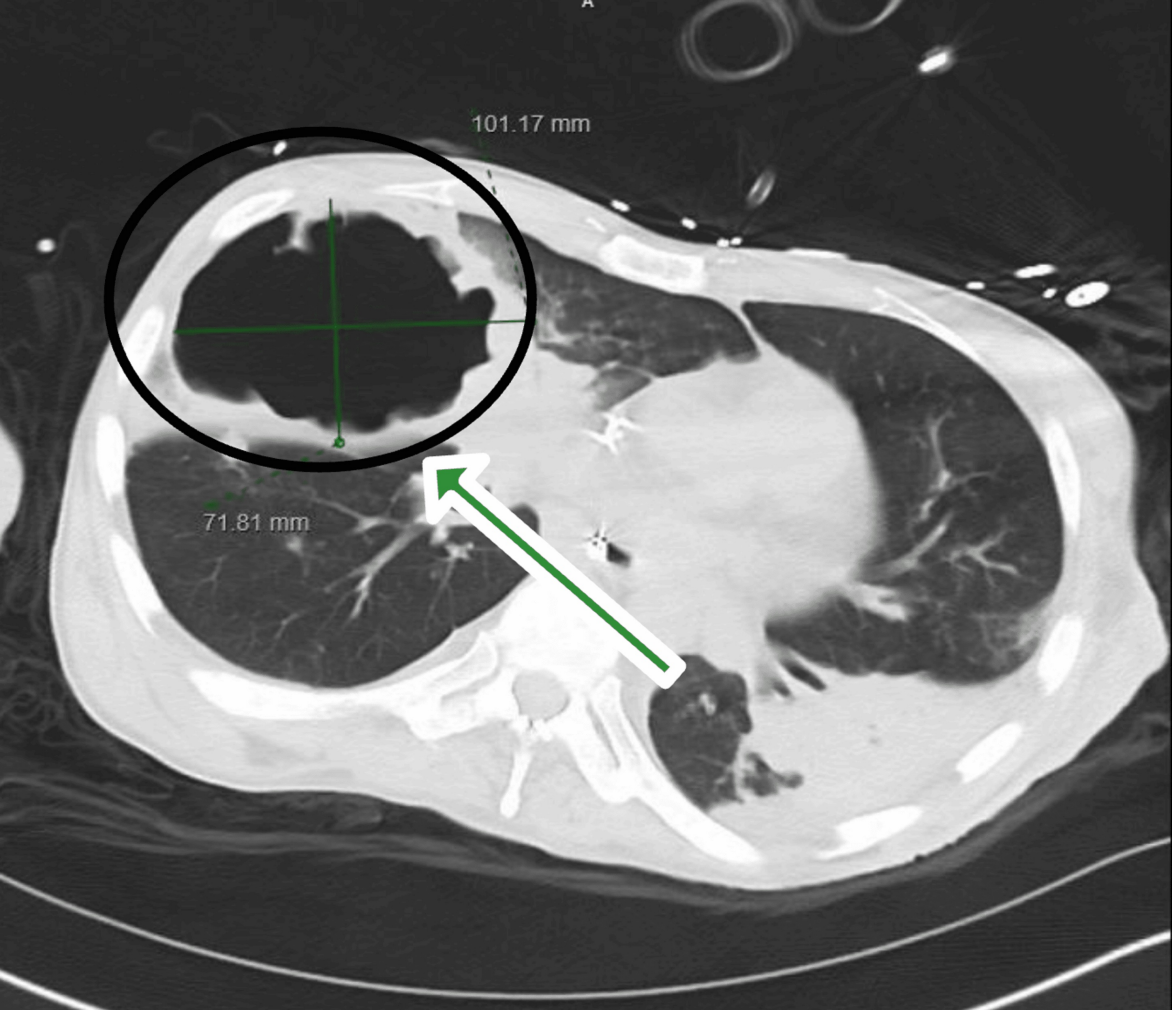
A contrast-enhanced computed tomography of the chest revealed multifocal airspace disease with a large cavitation in the right upper lobe measuring 10 x 7 x 12 cm with peripheral wall thickening and no internal fluid level (black circle with a green arrow)

Chest radiography revealed a large right-sided pulmonary cavitation measuring approximately 12 cm in diameter (Figure 1). Further imaging with contrast-enhanced computed tomography (CT) of the chest, abdomen, and pelvis showed multifocal airspace disease with a large cavitary lesion in the right upper lobe measuring 10 x 7 x 12 cm, with peripheral wall thickening and no internal fluid level. No other intra-abdominal or pelvic source of infection was identified (Figure 2).

Empiric broad-spectrum antimicrobial therapy was initiated, including vancomycin, meropenem, and anidulafungin, with an additional dose of tobramycin administered. Stress-dose corticosteroids were also started. Initial blood cultures revealed gram-negative bacilli, and the BioFire panel was positive for *S. marcescens* by PCR. Definitive blood cultures subsequently grew *Serratia marcescens* after 48 hours, at which time the patient had already expired. The isolate was resistant to ampicillin, ampicillin/sulbactam, aztreonam, cefazolin, ceftriaxone, and gentamicin (Table 1). Endotracheal aspirate cultures also grew *S. marcescens* with an identical resistance pattern (Table 2). In addition, urine cultures yielded *S. marcescens* with a similar resistance profile (Table 3).

**Table 1:** Blood culture with sensitivity results R: Resistant; S: susceptible; I: intermediate

Antibiotic	Result
Ampicillin	R
Ampicillin/Sulbactam	R
Aztreonam	R
Cefazolin	R
Cefepime	S
Ceftriaxone	R
Ciprofloxacin	I
Gentamicin	R
Meropenem	S
Piperacillin/Tazobactam	S
Tetracycline	I
Tobramycin	I
Trimethoprim/Sulfamethoxazole	S

**Table 2:** Endotracheal aspirate culture with sensitivity results R: Resistant; S: susceptible; I: intermediate

Antibiotic	Result
Ampicillin	R
Ampicillin/Sulbactam	R
Aztreonam	R
Cefazolin	R
Cefepime	S
Ceftriaxone	R
Ciprofloxacin	I
Gentamicin	R
Meropenem	S
Piperacillin/Tazobactam	S
Tetracycline	I
Tobramycin	I
Trimethoprim/Sulfamethoxazole	S

**Table 3:** Urine culture with sensitivity results R: Resistant; S: susceptible; I: intermediate

Antibiotic	Result
Ampicillin	R
Ampicillin/Sulbactam	R
Aztreonam	R
Cefazolin	R
Cefepime	S
Ceftriaxone	R
Ciprofloxacin	I
Gentamicin	R
Meropenem	S
Nitrofurantoin	R
Piperacillin/Tazobactam	S
Tetracycline	I
Tobramycin	R
Trimethoprim/Sulfamethoxazole	S

Due to worsening acute kidney injury and persistent metabolic acidosis, continuous renal replacement therapy was initiated. A comprehensive infectious workup including a respiratory viral panel and tuberculosis PCR was negative.

Despite aggressive supportive care and broad-spectrum antimicrobial therapy, the patient’s clinical condition deteriorated within 24 hours of presentation. Following discussions with the family and the palliative care team, his code status was changed to do-not-resuscitate (DNR), while mechanical ventilation was continued. The patient subsequently suffered a pulseless electrical activity cardiac arrest and expired within 24 hours. The cause of death was determined to be *S. marcescens* cavitary pneumonia complicated by severe septic shock.

## Discussion

*S. marcescens* is considered an opportunistic pathogen that typically causes serious infections in immunocompromised hosts, including those with advanced HIV. It can lead to a wide spectrum of diseases such as urinary tract infections, focal bronchopneumonia, endocarditis, and soft tissue infections [3]. Pulmonary infections due to *S. marcescens* often present with fever, cough, chest pain, and hemoptysis [4]. Radiologically, it may show focal bronchopneumonia, diffuse hematogenous infiltrates, lobar consolidation, and, in rare cases, microabscesses [5-7]

Pulmonary involvement is rare and typically occurs in immunocompromised patients. In the presence of pulmonary cavitary lesions, the differential diagnosis is broad and includes metastatic lung nodules, rheumatoid nodules, Langerhans cell histiocytosis, tuberculosis, Staphylococcus aureus pneumonia, mycotic infections, and septic emboli. Recognition of these imaging features, when integrated with clinical and laboratory findings, is essential for accurate diagnosis and timely management [7].

Although cavitary pneumonia due to *Serratia* is rarely reported [8], the formation of pulmonary cavities can result from various pathological processes, including suppurative necrosis (e.g., pyogenic abscess), caseous necrosis (e.g., tuberculosis), ischemic necrosis (e.g., pulmonary infarction), or cystic dilatation of lung structures (e.g., Pneumocystis jirovecii pneumonia). Malignancy-related cavities may also develop due to internal liquefaction or treatment-induced necrosis. Host factors, such as profound immunosuppression, and pathogen-specific mechanisms influence this tendency [9]. In HIV patients, *Serratia marcescens* infection is usually associated with low CD4+ counts of less than 70 cells/ml and an acquired immunodeficiency syndrome (AIDS) stage (as per CDC, AIDS or stage 3). (2) In our case, advanced immunosuppression due to HIV likely compromised pulmonary defenses, facilitating rapid bacterial proliferation. Furthermore, *Serratia’s* production of proteases may have contributed to lung tissue destruction, aligning with features of necrotizing pneumonia [10].

One of the organism’s notable characteristics is its occasional production of chromosomal AmpC β-lactamase, which can confer resistance to broad-spectrum β-lactam antibiotics and complicate treatment, as observed in our case [11]. However, *S. marcescens* is not among the most common AmpC-producing organisms, in contrast to classic HECKY organisms (Enterobacter species, Citrobacter species, and Klebsiella aerogenes). Additionally, *S. marcescens* possesses other virulence mechanisms, including the production of DNase, lipase, and gelatinase, as well as a pore-forming hemolysin (ShlA) that induces cytotoxicity and promotes inflammatory mediator release [12].

Biofilm formation, particularly on catheters and other indwelling devices, further contributes to antimicrobial resistance by limiting antibiotic penetration and promoting persistent infection. While these factors may complicate management, current evidence suggests that when antimicrobial susceptibility testing is available and demonstrates susceptibility, monotherapy with an appropriate agent, such as cefepime (given the risk of AmpC induction) or a carbapenem, is generally sufficient, as combination therapy has not been shown to improve outcomes over β-lactam or carbapenem monotherapy. Aminoglycosides may be considered in selected severe cases, and trimethoprim-sulfamethoxazole remains an option for uncomplicated urinary tract infections [12].

Cases have also been described in patients without traditional forms of immunosuppression. For instance, necrotizing pneumonia similar to our case has been reported in a 61-year-old man with severe asthma receiving dupilumab, an anti-IL-4/IL-13 monoclonal antibody, who developed bilateral cavitary lesions confirmed on computed tomography. Bronchoalveolar lavage cultures grew *Serratia marcescens*, and he required prolonged antibiotic therapy. Although patients receiving dupilumab are not considered profoundly immunocompromised, inhibition of IL-4/IL-13 signaling alters IgE-mediated and Th2 immune responses and may confer a modified immune state. This case highlights that *Serratia* should be considered in necrotizing pneumonia even in patients without classic immunosuppression, with certain biologic therapies potentially contributing to disease susceptibility or severity [13].

In the nosocomial setting, *S. marcescens* infections are frequently reported in intensive care and neonatal units, with isolates obtained from catheters, oxygenation devices, prefilled syringes, parenteral solutions, sinks, and even the hands of healthcare workers. The organism has been recovered from disinfectant solutions and double-distilled water, underscoring its metabolic versatility and ability to survive in adverse environments [1]. This environmental persistence increases the risk of outbreaks in healthcare settings, especially in cases of immunocompromised patients, and thus highlights the importance of strict infection prevention measures.

## Conclusions

This case highlights the severe and often fatal potential of *S. marcescens* infections in immunocompromised patients, particularly those with advanced HIV disease and multiple comorbidities. The development of cavitary pneumonia due to multidrug-resistant *S. marcescens* represents a rare but life-threatening complication that poses significant diagnostic and therapeutic challenges. Prompt recognition of this pathogen, early implementation of infection control measures, and rapid initiation of targeted antimicrobial therapy are critical to improving patient outcomes.
